# Inside OCD: Perspectives on the Value of Storytelling with Individuals with OCD and Family Members

**DOI:** 10.3390/healthcare9080920

**Published:** 2021-07-21

**Authors:** Jeffrey Pufahl, Jaison Nainaparampil, Carol A. Mathews

**Affiliations:** 1 Center for Arts in Medicine, University of Florida, Gainesville, FL 32611, USA; 2 Cornerstone Psychiatric Care, Palm Beach Gardens, FL 33410, USA; n@cp.care; 3 Department of Psychiatry and Center for OCD, Anxiety and Related Disorders, University of Florida, Gainesville, FL 32611, USA; carolmathews@ufl.edu

**Keywords:** storytelling, performance, OCD, mental health, arts in public health

## Abstract

The Center for Arts in Medicine at the University of Florida (UF) partnered with the UF Center for OCD, Anxiety, and Related Disorders to develop a storytelling program for individuals with obsessive compulsive disorder (OCD) and their families. Over ten weeks, participants shared stories regarding their experiences with OCD and engaged in theater and storytelling exercises. In collaboration with each other and the facilitators, participants workshopped and transformed their stories into a cohesive theatrical performance. Participants performed in front of a live audience and engaged in a post-show discussion with the audience, which focused on the diagnosis of OCD, stigma regarding the illness, and the benefits of the program. Program members participated in a post-program focus group and completed a qualitative and quantitative online survey. Participants reported improved understanding of their OCD, more acceptance from family and friends, less shame and guilt related to their OCD, and more confidence about sharing their OCD stories. Although the program was not designed to be therapeutic, participants also reported therapeutic value. Preliminary findings of this study suggest storytelling programs can lead to a reduction in both self-stigma and community stigma; improvement of understanding of the lived experience of OCD by families, loved ones, and clinicians; and facilitation of interpersonal connections.

## 1. Introduction

Obsessive compulsive disorder (OCD) affects one in fifty individuals worldwide and is a leading cause of disability among individuals aged 18–44 years, as well as being associated with profound stigma and shame [1]. Although treatment for this chronic psychiatric illness is generally effective [2], it rarely results in complete symptom remission [3,4]. Even after adequate treatment, evidence of persistent, albeit reduced, symptomatology is still common in individuals with OCD, leading to impairment for many, especially in social and work functioning. Additionally, family members of individuals with OCD experience considerable social and emotional burden due to reduction of social activities and increase in isolation and distress for both the person with OCD and their family members [5]. Two aspects of the lived experience of OCD that contribute to social and functional impairment are shame and stigma, particularly self-stigma [6]. Feelings of stigma and shame continue for many, even when obsessions and compulsions are minimal and under good control. Given the profound social implications of OCD and the therapeutic challenges to treating this condition, it is clear that additional approaches, including those focused on minimizing shame and stigma, are needed to maximize functioning and quality of life for affected individuals and their families.

One activity that shows promise both for individuals with OCD and their family/caregivers is group storytelling. Storytelling is an important method for people and communities to share knowledge and life experiences [7]. Through shared experience, stories infuse peoples’ lives with meaning, help validate their experiences, and connect them more deeply to themselves, others, and their communities [8]. In health care settings, storytelling can be used as qualitative research to gain insight into the personal experiences, health, and wellbeing of patients and caregivers [7,9,10,11,12,13,14,15,16]. Banks-Wallace (1998) identified six major functions of storytelling in groups: to provide (a) contextual grounding, (b) a means of bonding with others, (c) a means to validate and affirm experiences, (d) catharsis and venting, (e) a means for resisting oppression, and (f) a vehicle for educating others. There is growing evidence of the effectiveness of storytelling as a vehicle for public health education and research translation [17]. Storytelling performance, as qualitative research dissemination, can be a powerful tool to challenge dominant narratives and disrupt conventional ways of knowing (Gray, 2003) and has been used to educate the public about health-protecting practices, advocate for improved clinical care [18], and build awareness on a variety of health issues in communities [19,20,21].

Given the functions and benefits of storytelling programs, such a program has the potential to provide individuals with OCD and their families the opportunity to validate their experiences, bond with others who share these experiences, reduce the isolation these individuals experience, and perhaps provide solace when facing obsessions and compulsions. Participating individuals can also use this platform to help educate the public and de-stigmatize OCD through the telling of their stories. This paper describes one such storytelling program conducted in 2017/18 at the University of Florida (UF). The Center for Arts in Medicine (CAM) at UF partnered with the UF Center for OCD, Anxiety, and Related Disorders (COARD) and UF Performing Arts (UFPA) to develop a ten-week storytelling program specifically designed for individuals with OCD and their families. The goal of the program was to engage the participants in a structured storytelling experience that would result in a public performance and to evaluate the effects of the program among participants. Participants reported improved understanding of their OCD, more acceptance from family and friends, less shame and guilt related to their OCD, and more confidence about sharing their OCD stories. Although the program was not designed to be therapeutic, participants also reported therapeutic value. Preliminary findings of this study suggest that storytelling programs can lead to a reduction in both self-stigma and community stigma, improvement of understanding of the lived experience of OCD by families, loved ones, and clinicians, and facilitation of interpersonal connections.

## 2. Materials and Methods

### 2.1. Participant Recruitment and Selection

Participants were recruited through a variety of mechanisms, including direct referral by clinicians in the psychiatry and psychology clinics at UF or in the community, flyers posted in the community, and word of mouth. Participants were eligible to participate in the program if they (1) had obsessive compulsive disorder (OCD) or were a family member of someone with OCD, (2) were psychiatrically stable enough to participate in the program, as determined by the participating psychiatrists on the research team (CAM and COARD), and (3) were willing and able to attend the full ten-week program. Participants whose symptoms were deemed to be too severe for effective or safe participation were excluded. 

### 2.2. Program Description and Design

The program was offered free of charge, and the study was approved exempt by the UF IRB-2 (IRB#201703087) and was designed to include both quantitative and qualitative follow-up assessments of the participants following the performance. Attendance at the performance was assessed as an additional incidental outcome. The storytelling program was co-designed and directed by the first author and a professional coach with health counseling experience. Once a week for ten weeks participants met to share stories and learn the craft of storytelling. To provide added mental health safety, a psychiatric resident physician was selected to participate and offer stories from the clinical perspective, as well as provide a clinical presence in all sessions. Group members trained in improvisation and played theater games in order to develop performance skills, gain confidence, and build group trust. The instructional focus was technical, meaning participants were taught specific tools to develop, write, and perform stories. For example, storytellers explored how to begin and end stories, how to create and expand on tangents, and how to connect with an audience. Examples of weekly story prompts included: *aha! Moments*, *at my best*, and *at my worst*. Participants were first invited to share stories in response to prompts in sessions and then asked to work on the stories at home. Stories told in sessions were audio-recorded, transcribed, and emailed to participants to aid in their at-home writing and rehearsal process and then performed again the following week. Group members were invited to offer constructive criticism on story form and content and to reflect together on the meaning and relevance of the stories for those with and without OCD. In addition to the group work, peer-to-peer work on story content, form, and delivery was a key part of the process, giving participants opportunities to offer personalized advice and support. 

**Figure 1:**
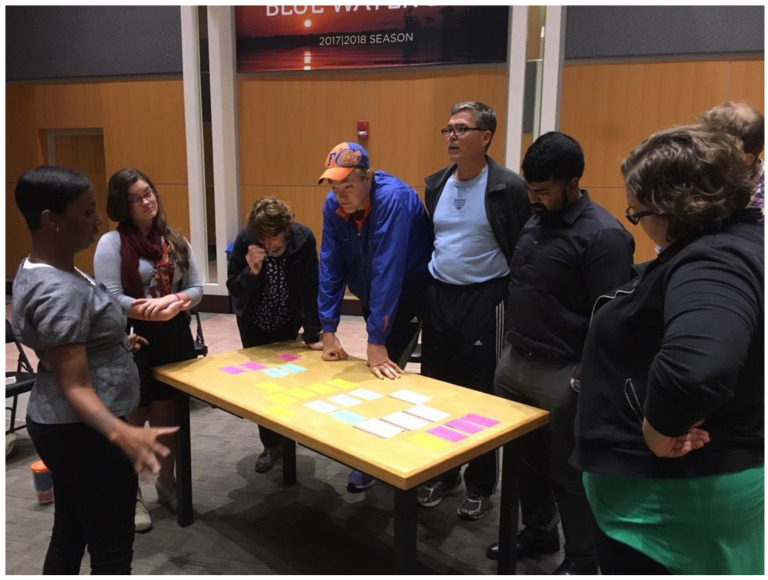
“Inside OCD” (obsessive compulsive disorder); participants and facilitators create the show through a process of collective dramaturgy involving note cards.

Once participants had selected and collaboratively shaped two to three stories each, the stories were distilled onto thematically color-coded index cards. The cards were then laid out on a table and together group members decided the running order of the stories (Figure 1). This method of collective dramaturgy allowed the group to control both the structure and content of the show and empowered them to develop the meta-story arc of the show. The program director then staged the stories in order to create a cohesive theatrical performance.

**Figure 2:**
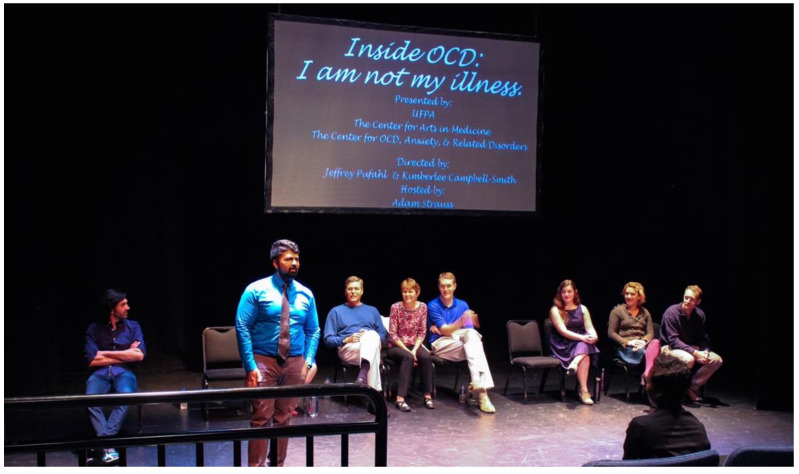
Participants perform “Inside OCD” at the University of Florida.

After staging and rehearsal, the show was performed, free to the public, at a performance space at the UF Performing Arts Center (Figure 2). After the performance, the audience was invited to engage in discussion with participants. The show was subsequently performed in 2018 at the International OCD Foundation (IOCDF) Annual Conference and at the 2019 American Psychiatric Association (APA) Annual Meeting; several of the stories were filmed and installed on the COARD website to allow for ongoing public access (See https://coard.psychiatry.ufl.edu/find-treatment/disorders-treatment/inside-ocd-i-am-not-my-illness/ (accessed on 15 July 2021).

### 2.3. Program Evaluation Methods

Following the UFPA performance, a focus group was conducted by a COARD clinician (C.A.M.) who had not participated in the storytelling program, and an online survey was administered via UF Qualtrics. The survey/focus group questions were developed by members of the research and facilitation team and were designed to assess (1) participants’ experiences and learning about their own OCD and (2) any therapeutic effect experienced by participants. The focus group consisted of eleven questions (see Table 1) and the online survey was composed of nine statements to which respondents answered on a five-point Likert scale ranging from strongly disagree to strongly agree (see Table 2). These statements were followed up with seven open-ended questions. 

The online survey was conducted anonymously through Qualtrics. The quantitative data were analyzed in Qualtrics. Data from the answers to the focus group questions were extracted and themes were analyzed by members of the CAM research team who were blinded to participants’ identities. Themes were identified in an iterative process. First, each response was read through and key content was highlighted. Second, the highlighted words/phrases across all responses were examined for similar themes and grouped accordingly. This process was repeated several weeks later and the results of the two processes were compared for consistency. Identified themes were compared to ensure non-overlap, and descriptive titles were derived for each identified theme. Finally, the themes, along with the keywords and phrases, followed by the entire sentence or response, were collated, and a second member of the research team (J.N.) examined them for consistency and relevance. Information about group members’ reasons for participating as well as their experience in the program (both positive and negative), and what they believed that they had achieved, if anything, was derived from qualitative examination of recurring themes extracted from transcripts of the post-performance focus group, and from the survey responses.

## 3. Results

### 3.1. Participants and Performance 

Twelve participants were recruited into the program; ten individuals with OCD and two parents of one individual with OCD. Eleven of the twelve participants were white. Participants ranged in age from 21–55 and came from a variety of educational backgrounds; most participants had an undergraduate degree or higher or were currently in university. The group was gender-balanced.

The majority of participants were referred by COARD clinicians, although three were recruited through community advertising. One participant dropped from the program before the third session; this participant noted that their symptoms felt too acute and that they were uncomfortable in the storytelling process. The remaining eleven participants completed the workshop and participated in the UFPA performance, which was attended by 175 individuals.

### 3.2. Quantitative data

**Figure 3:**
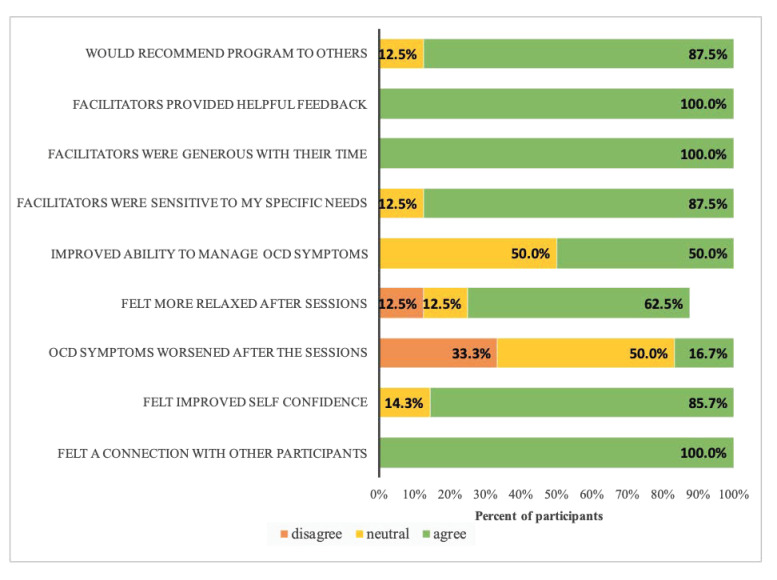
Results of the post-performance survey of “Inside OCD” group members (*n* = 8).

The post-performance online survey was completed by 8 of the 11 participants. Respondents included patients with OCD (*n* = 5), parent caregivers (*n* = 2), and one clinician (*n* = 1). The study group was gender-balanced with four members identifying as male, and four female. Survey responses were collapsed into three categories: “disagree”, “neutral”, and “agree.” As can be seen in Figure 3, response to the program was overwhelmingly positive, and 88% of participants reported feeling that they would recommend similar programs to others. Participants universally or nearly universally felt that the facilitators were generous with their time, attended to the specific needs of each individual, and provided helpful feedback. Feelings of increased social connection and increased self-confidence were also reported. Although not designed to be therapeutic in nature, about half of the participants also reported a therapeutic benefit, in particular, feeling more control over their OCD symptoms at the end of the process. Sixteen percent reported that their symptoms worsened after the sessions, while the rest either disagreed with or were neutral when responding to this item.

### 3.3. Qualitative data

The following recurring themes were identified from the focus group transcripts: reasons for participation, process, and outcomes (understanding and acceptance, connection and self-disclosure, creativity and confidence, and vulnerability). These themes are described in more detail below.

#### 3.3.1. Reasons for Participation 

Participants noted several reasons for choosing to participate in the storytelling workshop: the most frequently cited reason was feeling socially isolated and wanting ways to increase connections. Other reasons for agreeing to participate included wanting to find additional ways to target residual OCD symptoms, being at a transition point and looking for distractions or change, and wanting to change the way OCD is perceived. Almost all participants noted an interest in the creative aspect of the program, as well as wanting to share experiences with others who had lived experience of OCD.

#### 3.3.2. Process

Participants reported that the workshop and storytelling process was emotionally intense and time-consuming. Low self-confidence, feelings of vulnerability, and competing time commitments were noted by many, and all but one participant reported feelings of wanting to quit at one time or another. Stated reasons for staying primarily revolved around feeling a sense of responsibility to and respect for the facilitators and other participants. Over time, interest in participating evolved from the reasons described above to one universal theme: wanting to tell their individual stories, “wanting to get [my] story out”. At the end of the process, all participants but one reported being glad that they completed the workshop and the performance; that one participant was ambivalent on this point.

#### 3.3.3. Outcomes

Perceived outcomes included four key themes: (1) understanding and acceptance; (2) connection and self-disclosure; (3) creativity and confidence, and; (4) vulnerability.

#### 3.3.4. Understanding and Acceptance

Participants endorsed a better understanding of their OCD and increased confidence about sharing their “OCD stories”, gaining acceptance from family and friends, and feeling less shame and guilt related to their OCD diagnoses (Table 3). Because this program required participants to share otherwise untold stories or experiences related to their OCD, participants also reported feeling deeply connected to others with OCD for the first time.

#### 3.3.5. Connection and Self-Disclosure

Participants also reported that the program had therapeutic value, and improved their interpersonal relationships (Table 4). They reported an increased ability to laugh and to put their OCD symptoms in perspective, both in terms of their own life stories, and in terms of the larger lived experience of others.

One family member reported that a greater sense of family cohesion was achieved through the increased communication facilitated by the program.

*“It opened up more conversation in our family about the OCD and how it affects all of us.”*

#### 3.3.6. Creativity and Confidence

Group members also commented on the strength of the theatrical work and the benefits of the storytelling process and performance (Table 5). Many group members noted feeling connected to and supported by the facilitators. One, who had no prior storytelling experience, felt that she had found a new hobby, and another, who had prior experience, felt that telling stories that involved very personal, previously unrevealed information about OCD symptoms, improved her storytelling skills.

#### 3.3.7. Vulnerability

A few participants perceived some negative effects of the program, which highlights the need for professional mental health supervision and support for storytelling programs of this nature. One participant reported that their OCD symptoms transiently worsened immediately before or after a session, or that the sessions themselves were emotionally exhausting. One participant reported initial concern about how much to disclose, and two questioned the presence of a psychiatrist in training or family members during the sessions (Table 6).

## 4. Discussion

This pilot study is, to our knowledge, the first of its kind and adds to current literature that supports social integration as a significant determinant of health. The experience of participants in this storytelling program, as evidenced by both the qualitative and quantitative data, suggests that such programs can contribute positively to participants’ social support structures, relationships, and feelings of well-being by increasing feelings of confidence and social connectedness, as well as decreasing feelings of social isolation. While this was not designed to be a therapeutic program, we also found that storytelling may have therapeutic value for individuals with OCD. Despite some transient increase in symptoms or negative emotions immediately following the practice sessions, overall, participants reported feeling less burdened by their OCD and less alone. They also reported feeling more able and willing to self-disclose their OCD diagnoses, suggesting that the experience also resulted in decreased shame and self-stigma. Although not formally assessed, feedback from audience members for all three performances (e.g., community members, attendees at the IOCDF Annual Conference, and psychiatrists at the APA Annual Meeting) suggest that the performances were successful in increasing understanding of the lived experience of OCD, thereby potentially decreasing stigma. Formal audience assessment and further research into the true therapeutic nature of programs like this one are needed to fully understand the effects of storytelling on audience attitudes towards individuals with OCD. With participants reporting improved confidence and a better understanding of their illness, additional studies are needed to assess whether storytelling can aid in accelerating or improving outcomes for more standard therapeutic interventions. Further research into how the social isolation of those with OCD could be ameliorated by participating in similar programs also would be of benefit.

### Limitations

As this program was not designed to be therapeutic, we did not formally assess premorbid functioning or therapeutic outcomes. The sample size was relatively small, and thus our data require replication. The sample also consisted of a relatively restricted group of individuals, who were highly educated and predominantly white, limiting generalizability. Similarly, all participants had previously been in treatment for their OCD, possibly confounding the findings. Future endeavors should more formally assess both positive and negative potential therapeutic outcomes and should include a wider range of participants. In order to facilitate comparisons across participant groups, a formal study protocol should be developed.

## 5. Conclusions and Implications for Practice

This study provides information to assist clinicians in becoming aware of the utility of storytelling programs for their patients with OCD and perhaps also for those with other psychiatric illnesses. Patients who have had a reasonable response to treatment, and are sufficiently stable to tolerate what may at times be an emotionally intense experience, may find that programs such as the OCD storytelling program piloted at UF can help to decrease shame, stigma, and social isolation. For appropriate patients, collaboration with artists in this way may provide a sense of community and belonging, as well as encouragement to creatively examine their lived experiences of OCD.

Community artists seeking to work with individuals with mental health diagnoses and implement local storytelling programs should seek out partnerships with clinicians willing to collaborate and refer patients into programs. Mental health professionals can assess the readiness of individuals to participate, guide and assist artists in program design, and offer critical support during and after storytelling sessions.

## Figures and Tables

**Table 1:** Focus Group Questions.

Focus Group Questions
1. What was happening in your life at the time when you first heard about this experience?
2. What initially drew you to this project?
3. Why did you ultimately decide to participate in this project?
4. What did you hope to achieve by participating in the project? Did this evolve over the course of the project? If so, how?
5. Did you ever consider quitting? Why didn’t you quit?
6. What do you feel you have achieved by participating in this project?
7. How much control have you felt in the past over your OCD story? Has this shifted at all over the course of the project?
8. How has your confidence in telling your stories evolved over the past 10 weeks?
9. What have you learned about OCD as a result of participating in this project?
10. What did you want the audience to leave with after watching the show?
11. What new learning do you take away from this experience? How will you use what you’ve learned?

**Table 2:** Online Qualtrics Survey Questions.

On a Scale of 1–5 Where 1 Is “Strongly Disagree” and 5 Is “Strongly agree”, Please Indicate Your Level of Agreement with the Following Statements. If You Are a Family Member/Caregiver, Please Skip Questions Related to You Having OCD Symptoms.
1. I felt a connection with other members of the project.
2. After sessions, I felt more relaxed.
3. After sessions, my OCD symptoms would be worse.
4. I found this program improved my ability to manage my OCD symptoms.
5. I would recommend this project to other people with an OCD diagnosis.
6. I noticed an improvement in my self-confidence over the course of this project.
7. The facilitators were sensitive to my specific needs.
8. The facilitators were generous with their time.
9. The facilitators provided me with helpful feedback.
Open-ended questions:
10. How has participating in the project affected your OCD? Have you noticed any changes in your symptoms or your ability to manage your symptoms?
11. How have the dynamics of this group influenced you?
12. What have you learned about yourself as a result of participating in this project?
13. What changes have noticed within your interpersonal relationships during this project?
14. What might you recommend the facilitators change or adjust for a future project?
15. What did you like least about the project?
16. What did you like best about the project?
17. Is there anything else you’d like to share with us that has not been addressed on this survey?

**Table 3:** Participant open-ended survey responses.

*Participating in this project has helped my OCD in terms of knowing that there are other people struggling with it. Also, it helped me to identify my symptoms.*
*The project has given me an opportunity to gain perspective about how OCD has influenced me over time, especially in relation to decisions I’ve made about my life. That perspective has given me motivation to more actively challenge the ways in which OCD has limited me.*
*I was so inspired and moved by the group. I found enormous relief in finding that I am not alone, nor “crazy.” I had not really discussed my OCD diagnosis with other people. It was wonderful to find a community and to talk about OCD.*
*It … helped me identify some other issues that actually were part of, or deeply affected by, my OCD. It gave me a greater understanding of how much OCD is a part of my life, and yet how I am much more than it.*

**Table 4:** Participant open-ended survey responses.

*Sharing a challenging experience with this group has helped motivate me to work against my tendency to isolate myself.*
*It was really amazing to be a part of a group of people with a similar affliction, and I felt that we were all equally humbled by our own challenges yet amazed by each other...and that helped support amazement with ourselves. It was a great bonding experience.*
*The group dynamics made me feel less alone, and focusing on others’ stories helped me become less focused on myself and my OCD in a positive way.*
*I am more open with friends and loved ones about my OCD as a result of this project. I am more interested and more open to socializing.*
*I feel less afraid of people knowing about my diagnosis, and therefore feel I am more authentic.*
*I’ve become more confident in discussing my mental health with others and in being empathetic to people’s lives.*
*I have become a little more open to people.*

**Table 5:** Participant open-ended survey responses.

*I particularly liked transforming my/our struggles into something creative.*
*I loved hearing other people’s stories and creating a piece that could be performed with an interpretive but cohesive storyline.*
*I learned I could talk in front of a large group of people without passing out. … It gave me a sense of confidence to know I could do this.*
*I learned how to connect more with an audience and with people from other backgrounds.*
*I learned that I am actually a good storyteller.*
*Giving the project a storytelling rather than therapeutic focus allowed us to explore our OCD in ways we would otherwise not have had an opportunity.*

**Table 6:** Participant open-ended survey responses.

*I would advise…. more clarity in the beginning of the psychiatrist’s role. While I appreciate [their] presence and story perspectives …, it didn’t help to make the space feel safer [in the beginning].*
*I think it would be cool to have just OCD sufferers in the group and exclude caretakers.*
